# Spherical particles derived from TMV virions enhance the protective properties of the rabies vaccine

**DOI:** 10.1016/j.dib.2018.10.030

**Published:** 2018-10-12

**Authors:** N.A. Nikitin, I.N. Matveeva, E.A. Trifonova, N.M. Puhova, A. Ya Samuylenko, S.A. Gryn, J.G. Atabekov, O.V. Karpova

**Affiliations:** a Department of Virology, Lomonosov Moscow State University, 1/12 Leninskie gory, Moscow 119234, Russia; b All-Russian Scientific Research and Technological Institute of Biological Industry, VNITIBP 17, Moscow region, Shchelkovsky district 141142, Russia

**Keywords:** Rabies vaccine, Plant virus, Spherical particles, Adjuvant

## Abstract

In this study the ability of spherical particles (SPs) obtained from the tobacco mosaic virus (TMV) virions to enhance the immunogenic potential of the vaccine was evaluated. TMV SPs were shown to increase the protective properties of the widely used effective Russian adjuvant-free rabies vaccine, composed of killed rabies virions. The results of the NIH potency test showed enhancement of protectivity, that is comparable with the effect of the incomplete Freund׳s adjuvant on the same vaccine.

**Specifications table**

**Table:** 

**Subject area**	*Biology*
**More specific subject area**	*Vaccines, Plant Virology*
**Type of data**	*Table*
**How data was acquired**	*NIH potency test*
**Data format**	*Analyzed*
**Experimental factors**	*Spherical particles production by thermal transition of TMV. Virus inactivation test*
**Experimental features**	*Enhancement of protective potential of commercial vaccine by new candidate adjuvant – TMV SPs*
**Data source location**	*Department of Virology, Lomonosov Moscow State University,1–12 Leninskie gory, Moscow 119234, Russia*
**Data accessibility**	*Data are provided with this article*

**Value of the data**

● Here we shown the ability of a potent immunity enhancer - spherical particles (SPs) derived from tobacco mosaic virus (TMV) virions by the thermal remodelling - to increase the protectivity of the effective killed rabies vaccine Rabikan^™^. Rabikan (composed of the inactivated rabies virions) is the most widely used adjuvant-free veterinary rabies vaccine in Russian Federation, that for a long time demonstrate high efficacy.
● Our data demonstrate that TMV SPs have nearly the same immune stimulating potency as Freund׳s incomplete adjuvant. However unlike Freund׳s incomplete adjuvant TMV SPs do not cause local inflammatory reactions on laboratory animal and can be easily biodegraded [1]. As well TMV SPs have low cost of production and high biosafety due to the absence of common pathogens between mammals and plants [2].
● The results clearly point out, that TMV SPs should be considered as a promising safe candidate for a next generation adjuvant.

## 1 Data

This work is an essential step for evaluation of TMV SPs as effective safe adjuvant for vaccines development. Here the data of TMV SPs enhancement of widely used adjuvant-free rabies vaccine protectivity were shown by NIH potency test.

## 2 Experimental design, materials and methods

### 2.1 SP production

The detailed protocol for extraction and purification of TMV from the infected *Nicotiana tabacum* L. cv. Samsun leaves was described previously [3]. SP samples were prepared from TMV according to [4]. The morphology and size distribution of the obtained TMV spherical particles were subsequently analyzed by transmission electron microscopy and nanoparticle tracking analysis according to [5]. TMV SPs samples were 100 µg per dose of vaccine with average particle size 285 ± 4 nm.

### 2.2 Ethics statement

All experimental procedures with animals were performed according to European Communities guidelines [6], guide for the care and use of laboratory animals [7] and Ethical Code of the Russian Veterinarian recommended on the XIII Moscow International Veterinary Congress of Russian Small Animal Veterinary Association on April, 2005 (Moscow, Russia).

### 2.3 Virus inactivation test

The lyophilized vaccine Rabikan^™^ (Ser. No 132, FSE "SHCHELKOVO Biokombinat", Russia) was used as a basic rabies vaccine for comparing of the adjuvant potential. In order to confirm virus inactivation, 10 mice were injected intracerebrally with 0.03 ml of the reconstituted vaccine [8]. Animals were observed daily within the 14-days interval for detection of obvious rabies symptoms.

### 2.4 NIH potency test

The reference vaccine (national standard antirabic vaccine СТО-00494189-0042-2010, 1.8 IU/ml) and the test samples of vaccine adjusted with different adjuvants were serially diluted with Hank׳s medium up to 1:5, 1:25, 1:125, 1:625 and 1:3125 (see Table 1). According to the standard protocol [8], [9], each dilution was injected intraperitoneally into 16 BALB/c mice twice with a 7 day gap. Mice were distributed equally by sex. To evaluate the titre of the challenge virus standard (CVS-11), 3 groups of 10 animals were injected intracerebrally with 0.03 ml of the CVS, diluted to 1:10, 1:100, 1:1000. Mice were challenged intracerebrally with CVS dose of 5–50 LD_50_ in 0.03 ml, 7 days after the last immunization and observed for 14 days. Deaths after the day 5 post lethal challenge were counted, as well as mice alive though symptomatic on day 14. According to NIH protocol, mice dead before 5 days following challenge were recorded as non-specific death. The number of registered non specific death was removed from the total number during effective dose (ED_50_) calculation. Deaths during experimental period were recorded as specific and non-specific using fluorescent antibody test as confirmatory using brain samples. The relative vaccine potency was calculated by comparison of ED_50_ with the same value, calculated for the reference vaccine. ED_50_ value was calculated according to the Spearman-Kärber formula. To reduce the suffering and total number of sacrificed laboratory animals, all vaccine batches with different adjuvants were tested simultaneously with a single batch of the reference vaccine. Animals were euthanized as soon as severe rabies symptoms being noticed.

**Table 1:** Results of the NIH potency test.

Vaccine/adjuvant	Vaccine Volume (ml/injection)	Vaccine dilutions	N. mice	N. of mice 14 days after the control intracerebral infection with the reference strain CVS-11 (cerebral strain - challenge virus standard)		ED_50_	Relative Potency (IU/ml)
				Survived	Dead		
National Reference Vaccine	0.5	1:5	16	16	0	2,75	1,8
		1:25	16	14	2		
		1:125	16	11	5		
		1:625	16	9	7		
		1:3125	16	5	11		
Rabikan^ТМ^+PBS (Control)	0.5	1:5	16	16	0	2,66	1,47
		1:25	16	13	3		
		1:125	16	11	5		
		1:625	16	9	7		
		1:3125	16	4	12		
Rabikan^ТМ^+TМV SPs -100 μg /dose	0.5	1:5	16	16	0	2,8	1,99
		1:25	16	13	3		
		1:125	16	12	4		
		1:625	16	10	6		
		1:3125	16	5	11		
Rabikan^ТМ^+Incomplete Freund׳s adjuvant	0.1^a^	1:5	16	16	0	2,79	1,98
		1:25	16	13	3		
		1:125	16	12	4		
		1:625	16^b^	9	6		
		1:3125	16^b^	5	10		

a The final injected volume was 0.2 ml, as 0.1 ml of Freund׳s incomplete adjuvant (Sigma) was added to each vaccine dilution.

b One non-specific death.
